# Membrane‐acting biomimetic peptoids against visceral leishmaniasis

**DOI:** 10.1002/2211-5463.13562

**Published:** 2023-02-07

**Authors:** Vivek Kumar, Jennifer S. Lin, Natalia Molchanova, John A. Fortkort, Carolin Reckmann, Stefan Bräse, Håvard Jenssen, Annelise E. Barron, Archana Chugh

**Affiliations:** ^1^ Kusuma School of Biological Sciences Indian Institute of Technology Delhi India; ^2^ Department of Bioengineering Stanford University, Schools of Medicine and of Engineering CA USA; ^3^ The Molecular Foundry, Lawrence Berkeley National Laboratory CA USA; ^4^ Institute of Biological and Chemical Systems – Functional Molecular Systems (IBCS‐FMS) Karlsruhe Institute of Technology (KIT) Germany; ^5^ Department of Science and Environment Roskilde University Denmark

**Keywords:** antimicrobial, antiparasitic, leishmania, peptide mimetic, peptoid

## Abstract

Visceral leishmaniasis (VL) is among the most neglected tropical diseases in the world. Drug cell permeability is essential for killing the intracellular residing parasites responsible for VL, making cell‐permeating peptides a logical choice to address VL. Unfortunately, the limited biological stability of peptides restricts their usage. Sequence‐specific oligo‐*N*‐substituted glycines (‘peptoids’) are a class of peptide mimics that offers an excellent alternative to peptides in terms of ease of synthesis and good biostability. We tested peptoids against the parasite *Leishmania donovani* in both forms, that is, intracellular amastigotes and promastigotes. *N*‐alkyl hydrophobic chain addition (lipidation) and bromination of oligopeptoids yielded compounds with good antileishmanial activity against both forms, showing the promise of these antiparasitic peptoids as potential drug candidates to treat VL.

## Graphical abstract

Biomimetic peptoids have membrane rupturing activity on VL, causing species *L. donovani* inhibition within 4 h of treatment. Hydrophobic lipid tail attachment further aids in membrane attachment and thereby improves activity against both promastigote and intracellular amastigote forms.

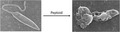

**Abbreviations**
LD: *Leishmania donovani*
LDH: lactate dehydrogenase
LPG: lipophosphoglycan
MTT: 3‐(4,5‐dimethylthiazol‐2‐yl)‐2,5‐diphenyl‐tetrazolium bromide
ROS: reactive oxygen species
PKDL: post‐kala‐azar dermal leishmaniasis
VL: visceral leishmaniasis

Visceral leishmaniasis (VL) is a neglected tropical disease that predominantly affects the poorest populations on Earth. According to WHO, VL accounts for 50 000–90 000 infections annually worldwide. However, the total number of VL infections is likely much larger, as it is estimated that just 25–45% of infections are reported to WHO (https://www.who.int/news‐room/fact‐sheets/detail/leishmaniasis). According to WHO's 2019 data, 90% of this disease is spread across only 10 countries of Southeast Asia, East Africa, and Brazil. Left untreated, death can result within 2 years of infection, during which time the patient also serves as reservoir for further spread of the disease [1]. VL is currently the deadliest parasitic disease aside from malaria [2]. It has been reported that both government and private institutions are showing less interest in tackling VL due to a decrease in the reported number of new infections [3]. However, this lack of interest is shortsighted, because it overlooks the full impact of this disease. This includes the increase of post‐kala‐azar dermal leishmaniasis (PKDL) and the co‐occurrence of VL with malaria [4] /HIV [5] and other similar pathological diseases that have further complicated the elimination program. PKDL, which occurs in VL‐cured patients after 6–24 months [6], acts as a reservoir for further infection and is responsible for the spread of the disease to nonendemic areas [7]. Climate change, war, and terrorism are also leading to a mass migration of disease‐infected people. These mass migrations may increase the spread of the disease drastically and may further delay reaching the disease elimination target of reducing cases to less than 1 in 10,000 in the population [8]. Above all, the emergence of COVID in India and other VL‐affected countries may augment the spread and emergence of this disease [9]. It is thus highly likely that elimination of the disease will become more challenging if appropriate novel strategies are not developed for its effective management in a timely manner.

Achievement of the disease elimination target is currently facing several hurdles. These include inherently long treatment regimens, high cost of available drugs, emergence of resistance to existing drugs, the failure of vaccines against VL in phase II and III clinical trials, and a current absence of alternative drugs for VL treatment [10, 11]. The condition is further exacerbated by an increase in demand for existing VL drugs as a result of their use in other indications. In particular, the COVID pandemic has resulted in an increase in the use of Amphotericin B/AmBisome for post‐COVID infections such as mucormycosis and a repurposing of AmBisome to Remdesivir for COVID treatment [3]. Thus, in order to reach sustainable development goal 3.3 for the year 2030 (elimination of VL) [8], there is an urgent need to develop a drug that is low cost, less susceptible to the emergence of resistance, and also effective against *Leishmania donovani*, the causal organism of VL. Consequently, potential drug candidates for the treatment of VL should have suitable permeability, making cell‐penetrating peptides good candidates for this application [12]. Cell‐penetrating peptides have an inherent ability to cross the cellular membrane and carry diverse macromolecules [13, 14], but their intracellular stability and bioavailability remains a concern [15]. Therefore, this need may be addressed through the development of peptide‐based or peptoid‐based therapeutics, which are known for their protease resistance and enhanced bioavailability [16].

Due to the intracellular nature of *L. donovani*, peptide‐based or peptoid‐based drugs should be tested against both promastigote and amastigote forms of the parasite [17]. In order to kill intramacrophagic amastigotes, potential drug candidates will typically need to cross the macrophagic membrane. The best‐suited candidates for this purpose are small nonhydrophobic cell‐permeating drug molecules, or cell‐penetrating peptides and their mimetics. While multiple peptide‐based therapeutic molecules have shown efficacy against *L. donovani* [18], a class of peptide mimetics known as peptoids could offer a better alternative for the development of an anti‐VL drug. Peptoids differ from peptides as side chains are attached to the amide nitrogen, while in peptides, side chain attachment occurs at the α‐carbon. As a result of this structural difference, peptoids often exhibit decreased susceptibility to proteolytic degradation and better stability, functional versatility, and bioavailability than their peptide counterparts [16, 19]. Peptoids have shown activity against various pathogens, including viruses, bacteria, fungus, and parasites [20, 21, 22, 23, 24, 25, 26, 27]. However, most of the parasite studies have been carried out against either *L. amazonensis* promastigotes [25, 28] or *L. mexicana* axenic amastigotes [29]. To the best of our knowledge, the present study is the first report to successfully assess the effect of peptoids against *L. donovani* in both forms (intracellular amastigotes and promastigotes). The effect of hydrophobic chain addition and bromination of peptoids on the anti‐VL activity against both forms of leishmaniasis is also shown. This study thus establishes peptoids as promising components of a potential new antileishmanial strategy.

## Materials and methods

### *Leishmania donovani* and cell lines

The Bob strain of *L. donovani* (promastigotes) was the generous gift of Prof. Amitabh Mukhopadhyay (Kusuma School of Biological Sciences, IIT Delhi, New Delhi, India). Bob cells were cultured in M199 complete media (Sigma‐Aldrich, St. Louis, MO, USA), supplemented with 50 μg·mL^−1^ gentamycin, and sodium bicarbonate, at 25 °C. Promastigote forms of *L. donovani* were subcultured every 3–4 days with complete media. Murine macrophage RAW 264.7 cells were procured from National Centre for Cell Science, Pune, India and were cultured in DMEM (Gibco, Thermo Fisher Scientific, Waltham, MA, USA) supplemented with 10% FBS (Gibco, Thermo Fisher Scientific) at 37 °C and 5% CO_2_.

### Peptoid design and synthesis

For an in‐depth discussion of the design of the TM peptoid library please see [20]. “TM” stands for “Tasha Molchanova”, which is the name of the organic chemist who synthesized and purified these peptoids.

### *In vivo* inhibition assay

Promastigotes of bob *L. donovani* were harvested in the log phase, and parasite inhibition was determined through 3‐(4,5‐dimethylthiazol‐2‐yl)‐2,5‐diphenyl‐tetrazolium bromide (MTT) methods using twofold dilution techniques [30]. Briefly, promastigote density of 2 × 10^6^ cells was taken, and different peptoid dilutions were added (0.187–3 μm) in a total volume of 100 μL. Percentage parasite viability was evaluated at 4 h at 25 °C using MTT (Sigma‐Aldrich). Parasite viability rate (%) = [OD of test sample/OD of untreated sample] × 100.

To evaluate the inhibitory effect of different peptoids on intracellular amastigotes, RAW 264.7 cells were infected with logarithmic phase promastigotes [30]. Briefly, RAW 264.7 cells (10^5^ cells/well) were seeded in 24‐well plates with round sterile coverslips and allowed to adhere to the coverslip for 24 h at 37 °C at 5% CO_2_ DMEM media supplemented with 10% FBS. Adherent macrophages were infected with *L. donovani* promastigotes at 1 : 10 (macrophage: promastigotes) for 6 h. Co‐infected cells were washed with PBS three times and incubated for a further 24 h for infection establishment. After 24 h, media were removed and again washed with FBS‐free DMEM followed by peptoid treatment. Ten μm of different peptoids were added to the co‐infected cells and incubated for 4 h at 37 °C at 5% CO_2_. After 4 h, peptoid‐treated co‐infected cells were washed with PBS, fixed with 100% methanol, and stained with diluted Giemsa stain (1 : 10) (HiMedia, Mumbai, India). The parasite clearance status of peptoids was determined by counting the number of the parasites using the Leica LAS core microscope system by counting 100 cells per coverslip.

### Scanning electron microscopy

Sample preparation for scanning electron microscopy was performed following this protocol [30]. Briefly, different peptoid‐treated (1–3 μm for 4 h) promastigote cells of bob *L. donovani* were fixed with 2–3% glutaraldehyde followed by gradual dehydration, air‐drying, and coating with 1% OsO_4_ and observed under scanning electron microscopy (Zeiss EVO 18 Special) at 20 kX magnifications.

### Quantification of lactate dehydrogenase release

Promastigote cell viability was quantified in terms of LDH release following a published protocol [30]. Briefly, promastigote cells of *L. donovani* were treated with varying concentrations (1–5 μm) of peptoids for 4 h in PBS at 25 °C; the reaction volume was 200 μL. LDH assay was performed using the LDH‐cytotoxicity assay kit according to the manufacturer's instructions (BioVision, Waltham, MA, USA).

### Quantification of reactive oxygen species

Reactive oxygen species (ROS) generated upon peptoid treatment (5 μm for 30 min) were quantified using a fluorescent dye cell‐permeant 2′,7′‐dichlorodihydrofluorescein diacetate (H_2_DCFDA) (Invitrogen, Thermo Fisher Scientific, Waltham, MA, USA) following a published protocol [30]. Fluorescence was measured using a fluorescence plate reader (Biotek Synergy H1) at Ex/Em of 495/529 nm. Data generated were converted into a logarithmic scale.

### Cytotoxicity assessment

The cytotoxicity of different peptoids against RAW 264.7 cells was assessed by the MTT method following a published protocol [30]. Briefly, murine macrophages were seeded in a 96‐well plate using DMEM supplemented with 10% FBS maintained at 37 °C and 5% CO_2_ for 24 h. Next, different concentrations of peptoids (5–80 μm) were added and incubated for 24 h followed by the addition of MTT for 2 h and solubilization of formazene crystals with DMSO addition. Percentage cell viability was calculated relative to the peptoid untreated sample.

### Statistical analysis

All experiments were carried out in triplicate and repeated at least three times independently (biological replicates), and the figures shown are representative of these experiments. IC_50_ was calculated using graphpad prism 6.0 software. The data were analyzed using Student's *t*‐test for comparison of two groups, and data values were expressed as mean ± standard deviation (SD). Significant differences were determined and designated with asterisks as follows: *P < 0.05; **P < 0.01; ***P < 0.001; and ****P < 0.0001.

## Results

In the present study, the antivisceral leishmaniasis activity of different peptoids was assessed. The effect on peptoid activity of bromination at one or more phenyl ring side chains and of the presence of a lipid chain of different lengths was also determined.

### *In vitro* antileishmanial activity

**Fig. 1:**
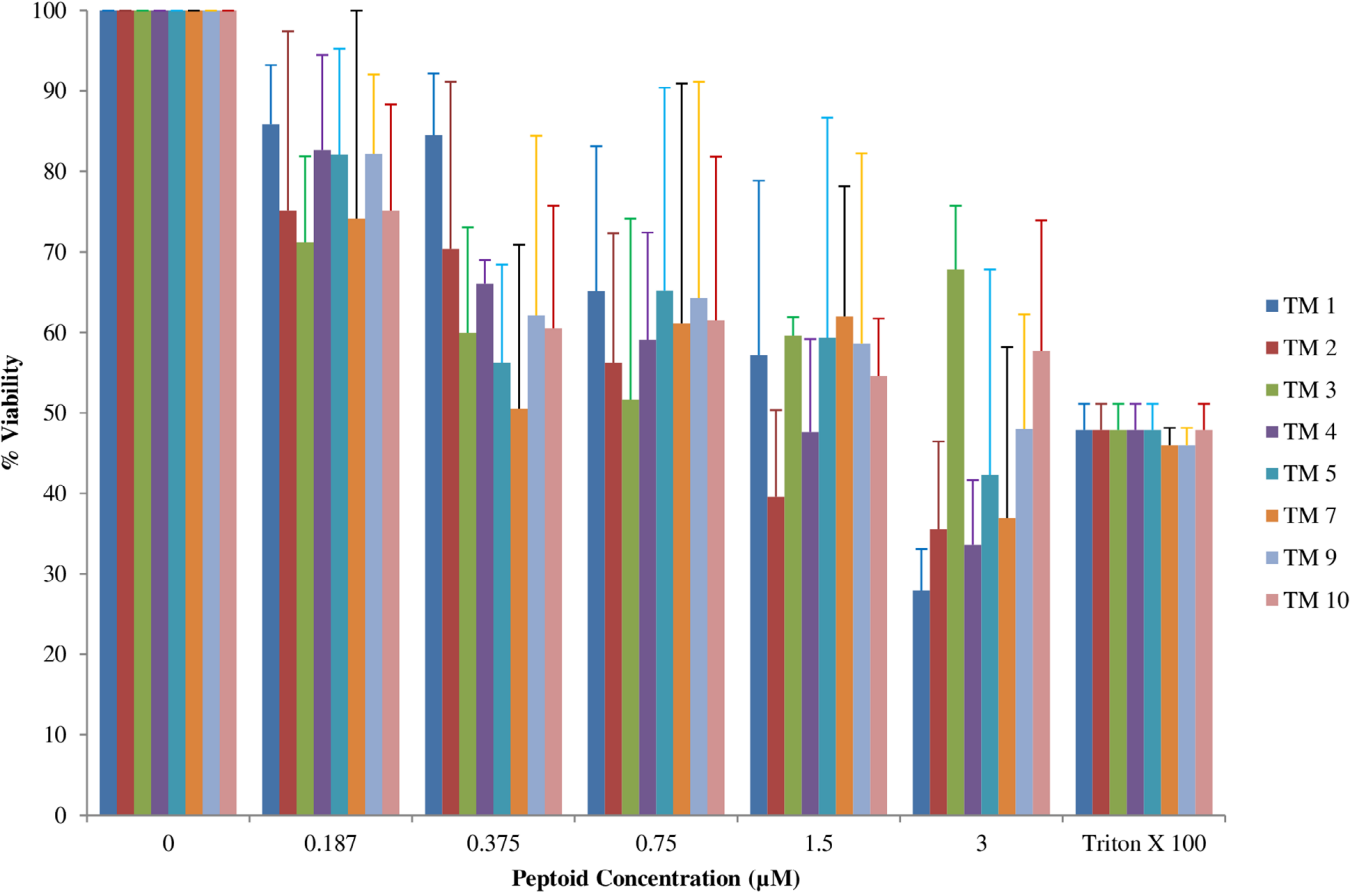
Antileishmanial activity of peptoids against *Leishmania donovani* promastigotes. Early‐logarithmic‐phase promastigotes of *L. donovani* bob strain were treated with different peptoids at different concentrations (0–3 μm) for 4 h. Negative control was considered 100% of cell viability (untreated) and positive control (Triton X treated). Cellular viability was assessed by MTT assay, and the IC_50_ values were calculated using graphpad prism 6.0. Error bars represent SD *N* = 3 and *n* = 3.

*Leishmania donovani* promastigotes were treated with a subset of the peptoid library reported previously [20] (TM 1, TM2, TM 3, TM 4, TM 5, TM 7, TM 9, and TM 10) with a range of concentrations (0.187–3.0 μm) for 4 h, followed by determination of parasite viability by MTT assay. All peptoids showed a range of inhibitory effects on promastigotes. Viability of promastigotes decreased with increasing concentration of peptoid in comparison with untreated cells (Fig. 1, Table S1). At 3 μm, peptoids TM1, TM 2, TM 4, TM 5, and TM 7 reduced promastigotes viability by 50% or more. Triton X 100 at 10% concentration was used as a positive control.

**Fig. 2:**
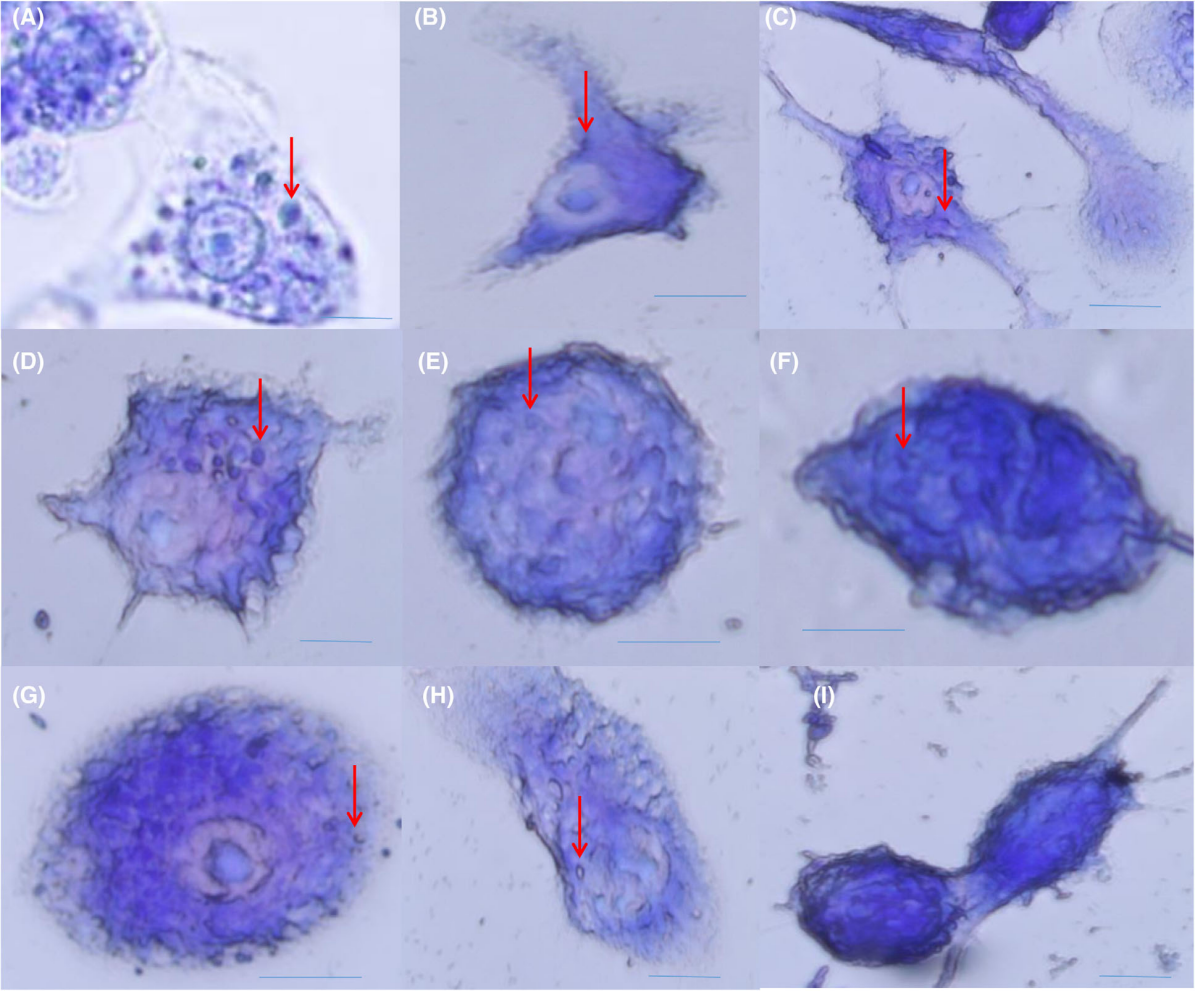
Giemsa staining of intracellular amastigotes in macrophage 264.7 cells after treatment with different peptoids at 10 μm concentrations. Untreated amastigotes (A), sample treated with TM 1 (B), TM 2 (C), TM 3 (D), TM 4 (E), TM 5 (F), TM 7 (G), TM 9 (H), and TM 10 (I). Red arrow points to the amastigotes form of *Leishmania donovani* parasite. Scale bar represents 10 μm.

Intracellular amastigotes were also treated with a subset of the peptoid library (TM 1, TM2, TM 3, TM 4, TM 5, TM 7, TM 9, and TM 10) at 10 μm concentration for 24 h followed by microscopy. Giemsa staining results indicated a reduction in number and size of amastigotes in samples treated with peptoid TM 1, TM 7, TM 9, and TM 10 compared with the control (untreated sample; Fig. 2). Based on the inhibitory effect of different peptoids on promastigotes, IC _50_ values of these peptoids were calculated (Table 1).

**Table 1:** Chemical and biological data of different peptoids used for the treatment of leishmanial parasite. +: Average, ++: Good, +++: Better, ++++: Best. *: Good, **: Better, ***: Best.

Peptoids	Structure	Sequences	Length (mer)	Promastigote IC _50_ (μm)	SEM of promastigote	Effect on intracellular amastigotes
TM1	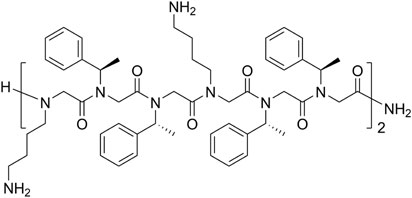	H‐(*N*Lys‐*N*spe‐*N*spe)_4_‐NH_2_	12	1.56	++	***
TM2	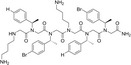	H‐(*N*Lys‐*N*spe‐*N*spe(p‐Br))_2_‐NH_2_	6	1.40	++++	*
TM3	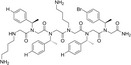	H‐*N*Lys‐*N*spe‐*N*spe‐*N*Lys‐*N*spe‐*N*spe(p‐Br) ‐NH_2_	6	4.60	+	*
TM4	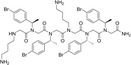	H‐(*N*Lys‐*N*spe(p‐Br)‐*N*spe(p‐Br))_2_‐NH_2_	6	1.38	++	*
TM5	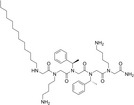	H‐*N*tridec‐*N*Lys‐*N*spe‐*N*spe‐*N*Lys‐NH_2_	5	2.35	+	
TM7	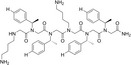	H‐(*N*Lys‐*N*spe‐*N*spe)_2_‐NH_2_	6	3.03	+++	**
TM9	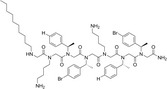	H‐*N*dec‐(*N*Lys‐*N*spe(*p*‐Br))_2_‐NH_2_	7	2.58	++++	***
TM10	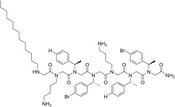	H‐*N*tridec‐(*N*Lys‐*N*spe(*p*‐Br))_2_‐NH_2_	7	1.54	++++	***

### Effects of peptoid treatment on *L. donovani* plasma membrane ultrastructure

**Fig. 3:**
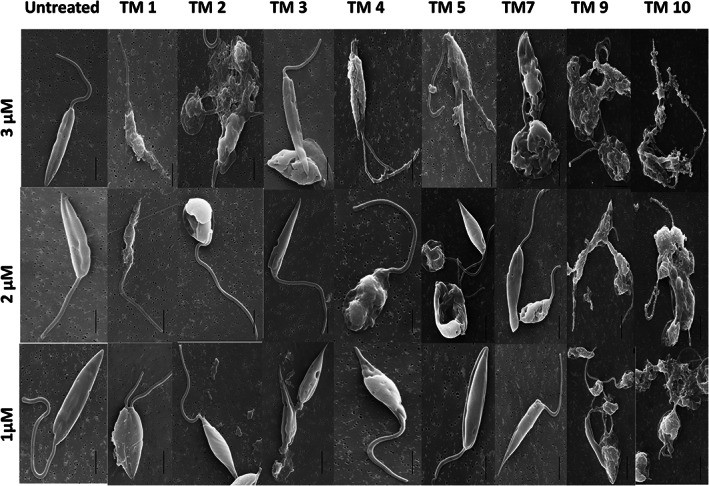
Ultrastructure of *Leishmania donovani* promastigotes upon treatment with a subset of peptoid library TM 1–10. Treatment time was 4 h. The left column shows typically untreated control promastigotes at different concentrations, followed in columns to the right by treatment with different TM peptoids, respectively, at three different concentrations. Scale bar represents 1 μm.

The tested peptoids induced significant morphological changes in *L. donovani* promastigote cells, including membrane weathering followed by shrinking, bursting, and aggregation of LD bodies (Fig. 3). These effects were observed to become more pronounced with increasing peptoid concentrations. Among all peptoids at 1 μm, TM 1, TM 9, and TM 10 exhibited visibly detectable effects, while at 3 μm, the morphological distortion of the LD body was observed for all peptoid‐treated samples (Fig. 3). The membrane damage further results in leakage of cytoplasmic content, leading to cellular constriction and bursting of the cell body.

### Assessment of the *L. donovani* promastigote plasma membrane upon peptoid treatment

**Fig. 4:**
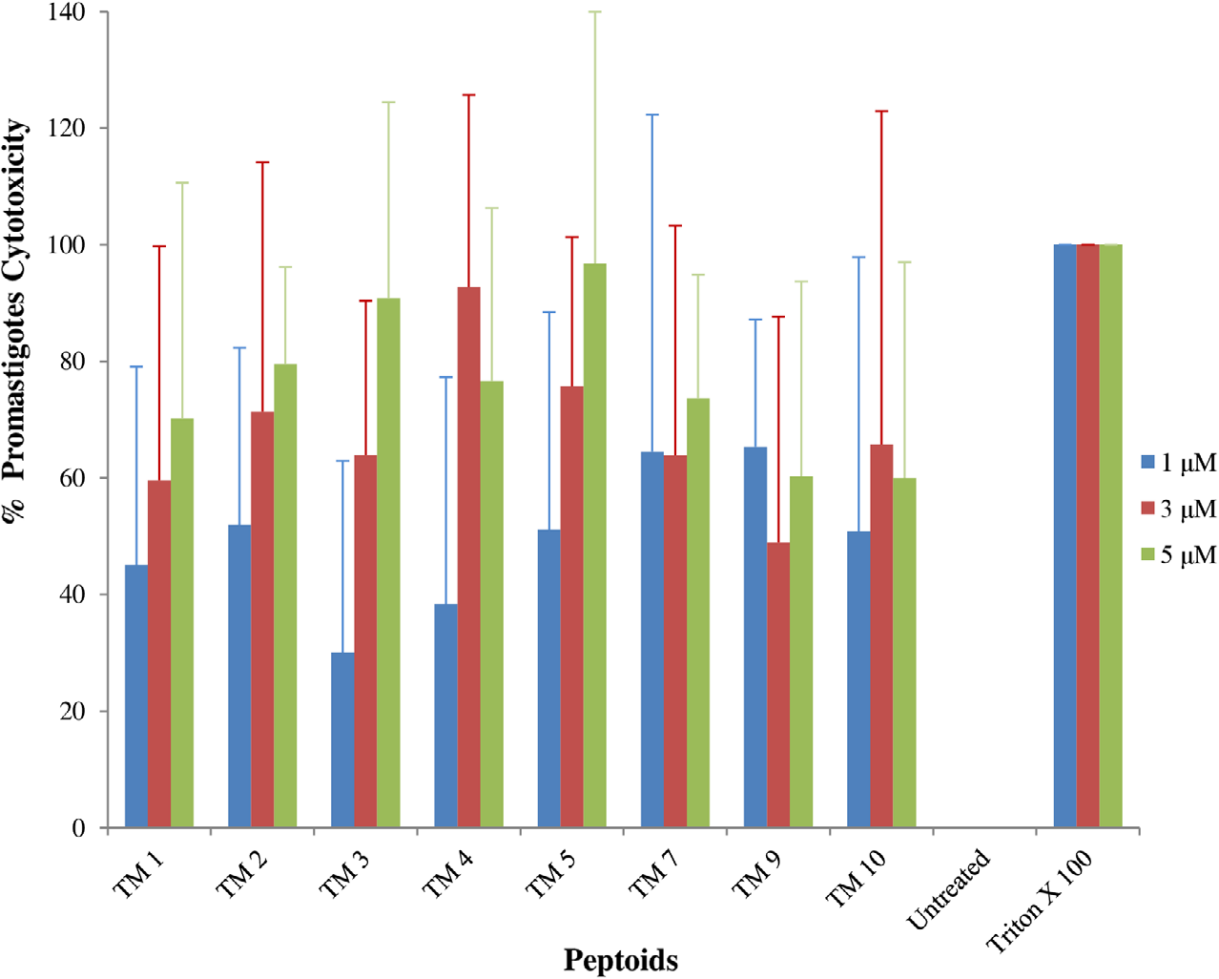
Percentage cytotoxicity of peptoids toward *Leishmania donovani* promastigotes as assessed in LDH release assay. Promastigote cells were treated with different concentrations of peptoids at 4 h. Percentage cytotoxicity was calculated as a measure of LDH release. Error bars represent SD *N* = 3 and *n* = 3.

Considering the apparent membrane‐attacking nature of these peptoids, the plasma membrane integrity of promastigotes was assessed by determining the release of lactate dehydrogenase enzyme (LDH) in the media. *L. donovani* cell viability was represented compared with untreated cells as the negative control vs Triton X 100 treated cells as the positive control. At 3 μm, almost all peptoids showed more than 60% cytotoxicity, increasing to 80% or more at 5 μm. At lower concentrations, peptoids TM 3 and TM 4 showed less than 40% cytotoxicity (Fig. 4).

### Assessment of intracellular ROS generation

**Fig. 5:**
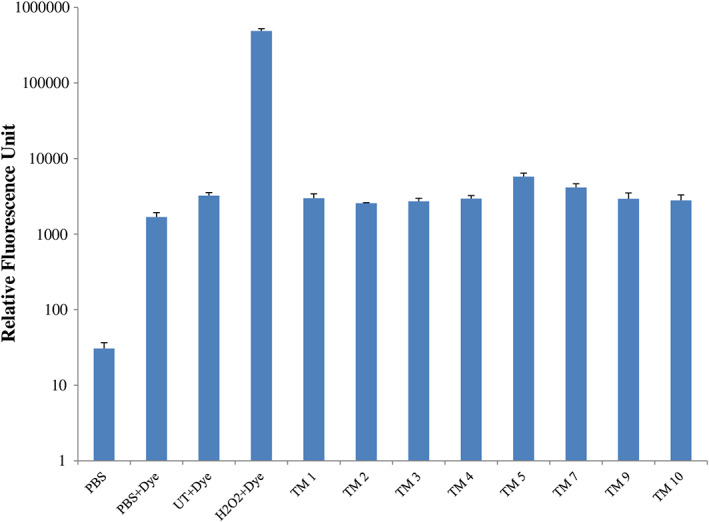
Peptoid induced ROS generation in *Leishmania donovani* promastigotes. No significant increase in ROS production compared with positive control (treated with H_2_O_2_). *Y* axis is represented in the logarithmic scale. Error bars represent SD *N* = 3 and *n* = 3.

Reactive oxygen species generation upon peptoid treatment of promastigotes of *L. donovani* was determined by fluorescent dye (H_2_DCFDA). The purpose of this experiment was to investigate whether these peptoids have an intracellular target. However, no significant increase in ROS generation was observed in any of the peptoid‐treated samples (Fig. 5).

### Effect of peptoids on murine macrophage cells

**Fig. 6:**
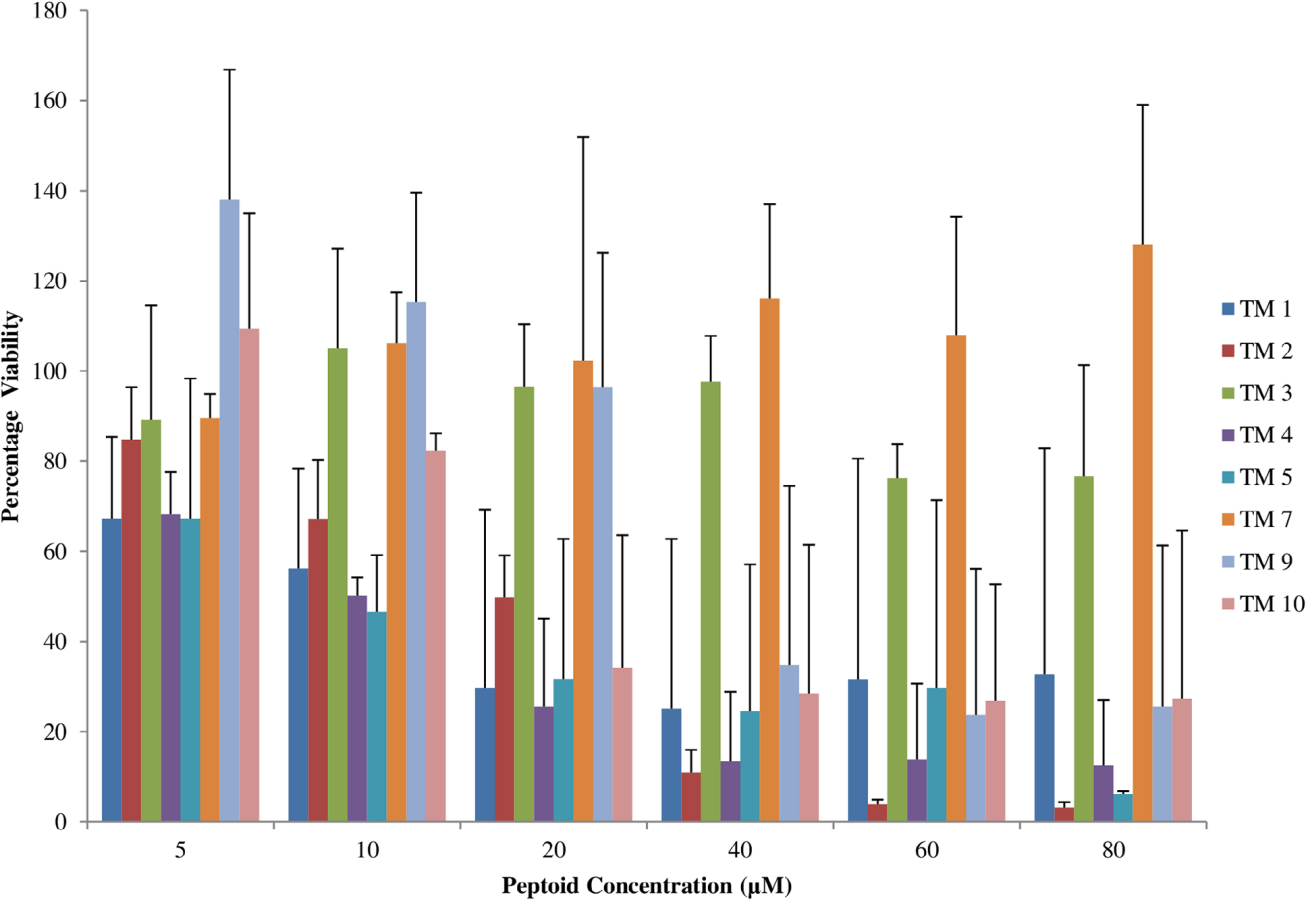
Percentage of living RAW 264.7 cells after incubation with different concentrations of peptoids. RAW 264.7 macrophage cells were incubated with peptoid for 24 h. Untreated cells (no peptoid treatment) showed 100% viable. The working concentration of 10 μm of peptoids caused only 50% or lower cytotoxicity in RAW 264.7 cells. Cell viability was determined by MTT assay, and data based on three experiments are represented by mean ± SD.

Cytotoxicity of different peptoids at a range of (5–80 μm) was determined on RAW 264.7 cells using an MTT assay. At the working concentration of 10 μm, peptoids TM 1 and TM 2 caused ~40% reduction in host cell viability and 50% viability reduction in the case of TM 4‐ and TM 5‐treated macrophagic cells, while peptoids TM 3, TM 7, TM 9, and TM 10 exhibited no significant reduction in cell viability. At 20 μm and above, drastic reduction in host cell viability was observed. Still, at 20 μm, peptoid TM 2‐treated cells showed more than 50% viability, and TM 3, TM 7, and TM 9 showed no toxicity against RAW 264.7 cells (Fig. 6, Table S2).

## Discussion

In this study, we report the effect of peptoids against *Leishmania donovani*, a causative species of VL. Peptoids were found to be active against both the promastigotes and intracellular amastigotes form of the parasite. Peptoids (other than TM 3) were found to be effective against *L. donovani* promastigote. Further, TM 2, TM 9, and TM 10 were found to be more effective among all peptoids tested (Figs 1 and 2). In particular, TM 9 and TM 10 emerged as the best peptoids against both promastigote and amastigote forms of the parasite (Table 1) while exerting no significant toxicity at a working concentration of 10 μm. These results suggest that out of all the peptoid design combinations that were tested, substitution of two bromine in the para position of two of the four phenyl rings appears to have increased the antileishmanial activity without significantly increasing host cell toxicity. At the same time, a decrease or increase in the degree of bromination (i.e., bromine substitution in one or all four of the phenyl rings) was found to have a deleterious effect on both antileishmanial activity and host cell toxicity (Table 1). It was also found that the addition of two bromine atoms along with the lipidation *N*dec (i.e., C10 tail; TM 9) or *N*tridec (i.e., C13 tail; TM 10) further improved the antileishmanial activity against both promastigote and amastigotes forms of the parasite with no significant increase in cytotoxicity. However, *N*tridec alone (no bromine addition) as in the case of TM 5 decreases antileishmanial activity and increases the host cell toxicity. Thus, for antileishmaniasis activity and host cell cytotoxicity, two bromine atoms along with an *N*‐alkyl hydrophobic tail within the peptoid structure are optimal. The most novel aspect of this study is that these peptoids exhibit antileishmanial activity after only 4 h of incubation, whereas the inhibitory action is normally observed between 48 and 72 h. In light of this, the antileishmanial activity of peptoid is significantly superior to that of existing similar peptoid and peptide mimetics. In terms of resistance development, the shorter treatment time has advantages over other developed peptoids.

Based on the literature, halogenation of antimicrobial peptides or peptoids enhances their antimicrobial efficacy by several fold without altering their hemolytic and cytotoxic properties [29, 31] and we observed a similar result in our study. Among halogens, addition of bromine to the side chain had the greatest effect in augmenting the antimicrobial efficacy of both peptides and peptoids [32]. Therefore, we focused on bromine substitution in our study. Previously, [29] investigated halogenated peptoid analogs for the antileishmanial activity against axenic amastigotes and promastigotes of *L. mexicana*. Although the peptoids with shorter length (9mer) lost their inhibitory activity against *L. mexicana*, the chloro substitution in the *para* position of the phenyl ring of peptoid retained its activity against axenic amastigotes but only at longer chain length > 9mer. In similar fashion, at 48 h of incubation, shorter peptoids containing fluorine substituted at the *para* or *meta* position also lost their inhibitory effect against axenic amastigotes but retained some activity for the 12mer length. Interestingly, in our study, we see efficacy against *Leishmania donovani* with bromine‐substituted peptoids that are 6 or 7mer in length. This may be due to the use of bromine instead of chlorine or fluorine for halogen substitution.

The antimicrobial activity of these peptoids has been investigated against several other organisms including bacteria [33] and viruses [20]. It is reported that these peptoids have both membrane‐attacking and intracellular modes of action [21]. In the present study, using scanning electron microscopy, it was observed that in *L. donovani*, these peptoids start attaching to the parasitic membrane, where they cause membrane instability (surface weathering) followed by the formation of pore‐like structures. This leads to morphological deformation and death (Fig. 3). However, parasitic death cannot be inferred from a mere change in surface morphology. Thus, the parasite killing of the peptoids was validated using an MTT assay and LDH release assays. LDH is released only when the cell membrane is compromised. It was observed that for each peptoid, the release of LDH starts at a treatment of 1 μm concentration and increases with increasing peptoid concentration (3 μm; Fig. 4).

Further increases in peptoid concentration increase the LDH release (Fig. 4), suggesting that increases in peptoid concentration will follow the same trend as the MTT result for *L. donovani* promastigotes. Apart from the cellular membrane, these peptoids could also have intracellular targets. In the case with prokaryotes, ribosome aggregation was observed [21]. Since VL‐causing parasites are eukaryotic [34], ribosomal aggregation‐based killing cannot be the mode of action of these peptoids. Other than ribosomes, mitochondria are the primary organelle affected by drug treatment. Mitochondrial damage leads to the instantaneous release of ROS [35]. In this report, the absence of a significant increase in ROS (Fig. 5) is the third indication that these peptoids have only a membrane disruption mechanism of antileishmaniasis. The two other indications were MTT assay and LDH release assays. However, other cellular targets need to be evaluated. In the present study, the *L. donovani* membrane appears to be an important site of action for the peptoids investigated. In the literature, these peptoids are known to have a high binding affinity toward lipopolysaccharide [36]. At the working concentration (i.e., at 10 μm), TM 3, TM 7, TM 9, and TM 10 act preferentially on the promastigote membrane, which is composed of polysaccharide lipophosphoglycan (LPG), but do not act on the host cell membrane whose outer surface carries phosphatidylcholine and sphingomyelin. It is also reported that leishmanial cell membranes comprise ergosterol, while mammalian cell membranes instead comprise cholesterol [37, 38]. Thus, it may be speculated that LPG or ergosterol could be the site of action in promastigotes while TM 1, TM 2, TM 4, and TM 5 may have another site of action that needs to be explored. With intracellular amastigotes, peptoids TM 7, TM 9, and TM 10 significantly reduced their count. Considering their membrane‐acting nature, these peptoids may have glycoinositolphospholipids as a target, which are specifically present in the amastigote membrane. Since TM 1 reduces the amastigote count and simultaneously and reduces the cellular viability by 40%, this peptoid may have a nonglycoinositolphospholipids target.

In previous studies, hydrophobic side chains and halogenations improved the antimicrobial activity and reduced the host cytotoxicity of peptoids [32, 39]. Considering the antileishmanial effect of TM 9 and TM 10, it can be summarized that lipidation with *N*dec and *N*tridec does indeed help peptoid TM 9 and TM 10 in parasite membrane attachment, while bromination augments antileishmanial activity. Further study of other peptoid combinations is warranted for a clearer picture of the optimal role of bromination for this application.

## Conclusions

In this study, we have shown that a particular class of peptide mimetics (peptoids) reduces the viability of *L. donovani*. To the best of our knowledge, this is the first study demonstrating the activity of peptoids against the *L. donovani* species of VL. The effect of bromination on antileishmanial activity, in terms of both number and position, has also been distinctly observed. At the same time, it has also been shown that the addition of a hydrophobic lipid tail (*N*dec and *N*tridec) improves the peptoid attachment to the parasite cell membrane. The peptoids in the present study have been shown to be effective against both promastigote and intracellular amastigote forms of *L. donovani* and thus could serve as the basis of a potential anti‐VL strategy.

## Conflict of interest

The authors declare no conflict of interest.

## Author contributions

VK, AEB, and AC conceptualized the project. VK performed the experiments and interpreted the data under the supervision of AC, JSL, NM, HJ, and AEB designed the peptoids. NM and CR synthesized the peptoids. SB mentored CR, VK wrote the manuscript. JSL, NM, and JAF contributed detailed information on peptoids. All authors edited the article.

## Supporting information


**Table S1.** Antileishmanial activity of peptoids against *Leishmania donovani* promastigotes.
**Table S2.** Host cell (RAW 264.7) Viability data.

## Data Availability

The data that support the findings of this study may be accessed as supplementary information or can be made available from the corresponding author on reasonable request.
