# Supplementary material for: Membrane‐acting biomimetic peptoids against visceral leishmaniasis
Source: FEBS Open Bio. 2023 Feb 7;13(3):519–31. doi: 10.1002/2211-5463.13562 (PMC9989931; doi:10.1002/2211-5463.13562)
Supplement: Supplementary file 1 — Table S1. Antileishmanial activity of peptoids against Leishmania donovani promastigotes. Table S2. Host cell (RAW 264.7) Viability data. [file FEB4-13-519-s001.docx]

**Table S1. Anti-leishmanial activity of peptoids against *Leishmania donovani* promastigotes**

**Table S2. Host cell (RAW 264.7) Viability data**
